# Inflammasomes as regulators of mechano-immunity

**DOI:** 10.1038/s44319-023-00008-2

**Published:** 2023-12-15

**Authors:** Jelena S Bezbradica, Clare E Bryant

**Affiliations:** 1 The Kennedy Institute of Rheumatology, University of Oxford, Oxford, UK; 2 Department of Veterinary Medicine, University of Cambridge, Cambridge, Cambridgeshire UK

**Keywords:** Inflammasomes, Mechano-Sensing, Mechano-Responses, Macrophages, Foreign Body Reaction, Cell Adhesion, Polarity & Cytoskeleton, Immunology, Signal Transduction

## Abstract

Mechano-immunity, the intersection between cellular or tissue mechanics and immune cell function, is emerging as an important factor in many inflammatory diseases. Mechano-sensing defines how cells detect mechanical changes in their environment. Mechano-response defines how cells adapt to such changes, e.g. form synapses, signal or migrate. Inflammasomes are intracellular immune sensors that detect changes in tissue and cell homoeostasis during infection or injury. We and others recently found that mechano-sensing of tissue topology (swollen tissue), topography (presence and distribution of foreign solid implant) or biomechanics (stiffness), alters inflammasome activity. Once activated, inflammasomes induce the secretion of inflammatory cytokines, but also change cellular mechanical properties, which influence how cells move, change their shape, and interact with other cells. When overactive, inflammasomes lead to chronic inflammation. This clearly places inflammasomes as important players in mechano-immunity. Here, we discuss a model whereby inflammasomes integrate pathogen- and tissue-injury signals, with changes in tissue mechanics, to shape the downstream inflammatory responses and allow cell and tissue mechano-adaptation. We will review the emerging evidence that supports this model.

## Abstract (web summary)

Mechano-sensing of tissue topology, topography or biomechanics alters inflammasome activity. This review discusses how inflammasomes integrate pathogen- and tissue-injury signals with changes in tissue mechanics, to shape downstream inflammatory responses and cellular mechano-adaptation.

## Graphical abstract

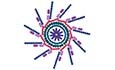

## Introduction: mechano-regulation of immunity

Mechanotransduction is the term that defines a process by which cells, including immune cells, convert sensing of changes in tissue mechanics into a signalling message and downstream response (Du et al, 2023; Lee et al, 2022). Mechanical cues include changes in tissue stiffness (e.g. caused by extracellular matrix changes), changes in topography/adhesive geometry (e.g. caused by the insertion of solid implants), or external load/forces (e.g. caused by the change in hydrostatic pressure, shear stress, tension or compression) (Du et al, 2023; Meli et al, 2019). Downstream responses to these mechanical cues involve intrinsic cell mechano-responses, such as a change in motility and cell adhesion (e.g. due to integrin adhesion), a change in cell shape and swelling (e.g. due to ion channel activity), a change in signalling or altered gene expression (Meli et al, 2019). The result is cell and tissue mechano-adaptation to maintain homoeostasis, but if dysregulated, chronic inflammation can occur. The mechanism by which cells sense and respond to mechanic forces to maintain immune homoeostasis and how they contribute to whole tissue mechano-inflammation are unanswered fundamental biological questions.

Mechano-sensing can occur between the two cells or between a cell and its tissue environment (for example, the extracellular matrix). One example where mechano-sensing occurs between the two cells is during the formation of the immune synapse. The immune synapse is a cell–cell interaction zone formed typically between a sensor cell, like a dendritic cell or a macrophage, and an effector cell, like a T cell, a B cell, or a natural killer cell. Within the immune synapse, cytoskeletal forces are generated on a nanoscale with immune receptors and integrins being recruited and spatially reorganised to coordinate the exchange of information between the interacting cells. The mechanical forces generated within the synapse such as those defined by the strength of receptor–ligand interaction affect the efficiency of immune receptor signalling, the duration of cell–cell contact, and the type of effector response generated (Upadhyaya, 2017).

A different example of mechano-sensing in immunity is between a cell and its tissue environment, such as macrophage sensing of tissue damage and infection. Macrophages are sentinels of tissue homoeostasis and are present in every tissue of the body. Our current understanding of how macrophages sense tissue signals to report the presence of tissue damage or infection is largely based on studying macrophage activation in vitro in response to soluble signals including damage- or pathogen-associated molecular patterns. Most inflammatory diseases where macrophages contribute to either pathology and/or resolution, however, also involve changes in biophysical properties of the tissue; one common example is tissue swelling. Cells of the monocyte/macrophage lineage, like many immune cells, will also encounter environments with diverse mechanical properties as they develop and migrate. These cells develop in the bone marrow, egress into the blood and lymphatics, and cross a variety of organs before differentiating at their final site of residence such that monocyte/macrophages can encounter a stiffness range from <1 to 70 kPa (Lee et al, 2022). The concept of how changes in tissue mechanics influence macrophage biology is only beginning to emerge.

One family of innate sensors that macrophages use to detect tissue damage and pathogen invasion are inflammasomes (Lamkanfi and Dixit, 2014). These cytosolic sensors can detect cellular invasion directly, for example, the NAIP/NLRC4 complex detects the presence of bacterial flagellin in the cytosol. Some inflammasomes detect cell damage indirectly, for example, NLRP3 detects changes in membrane integrity or composition and the loss of cytosolic ions (Lamkanfi and Dixit, 2014). Most inflammasomes use the adaptor ASC to assemble a macromolecular signalling platform, called a ‘speck’, where the effector enzyme caspase-1 becomes activated. Active caspase-1 cleaves two pro-inflammatory cytokines interleukin (IL)-1β and IL-18 into their active forms and cleaves the membrane pore-forming protein gasdermin D (GSDMD) allowing the release of IL-1β and IL-18. If it is not repaired, GSDMD pore formation results in cell lysis (pyroptosis) through the activation of the protein Ninjurin-1 (NINJ1), releasing many cytoplasmic proteins that serve as alarmins (Broz and Dixit, 2016; Kayagaki et al, 2021). Inflammasome activity is generally transient and beneficial in antimicrobial defence. Sustained inflammasome activity, in contrast, contributes to immuno-pathology in many chronic inflammatory conditions, for example in atherosclerosis, Parkinson’s disease, Alzheimer’s disease or during uncontrolled infections (Duewell et al, 2010; Gordon et al, 2018; Heneka et al, 2013; Lamkanfi and Dixit, 2014; Youm et al, 2013). We (Barone et al, 2022; Escolano et al, 2021) and others (Compan et al, 2012; Green et al, 2020; Ip and Medzhitov, 2015; Ran et al, 2023; Malik et al, 2011; Christo et al, 2016; Vasconcelos et al, 2022) recently described that inflammasome activity is regulated by changes in tissue mechanical properties and that sustained activity drives tissue mechano-inflammation and fibrosis (Barone et al, 2022; Vasconcelos et al, 2022).

## Inflammasome activity is regulated by mechano-sensing

**Figure 1:**
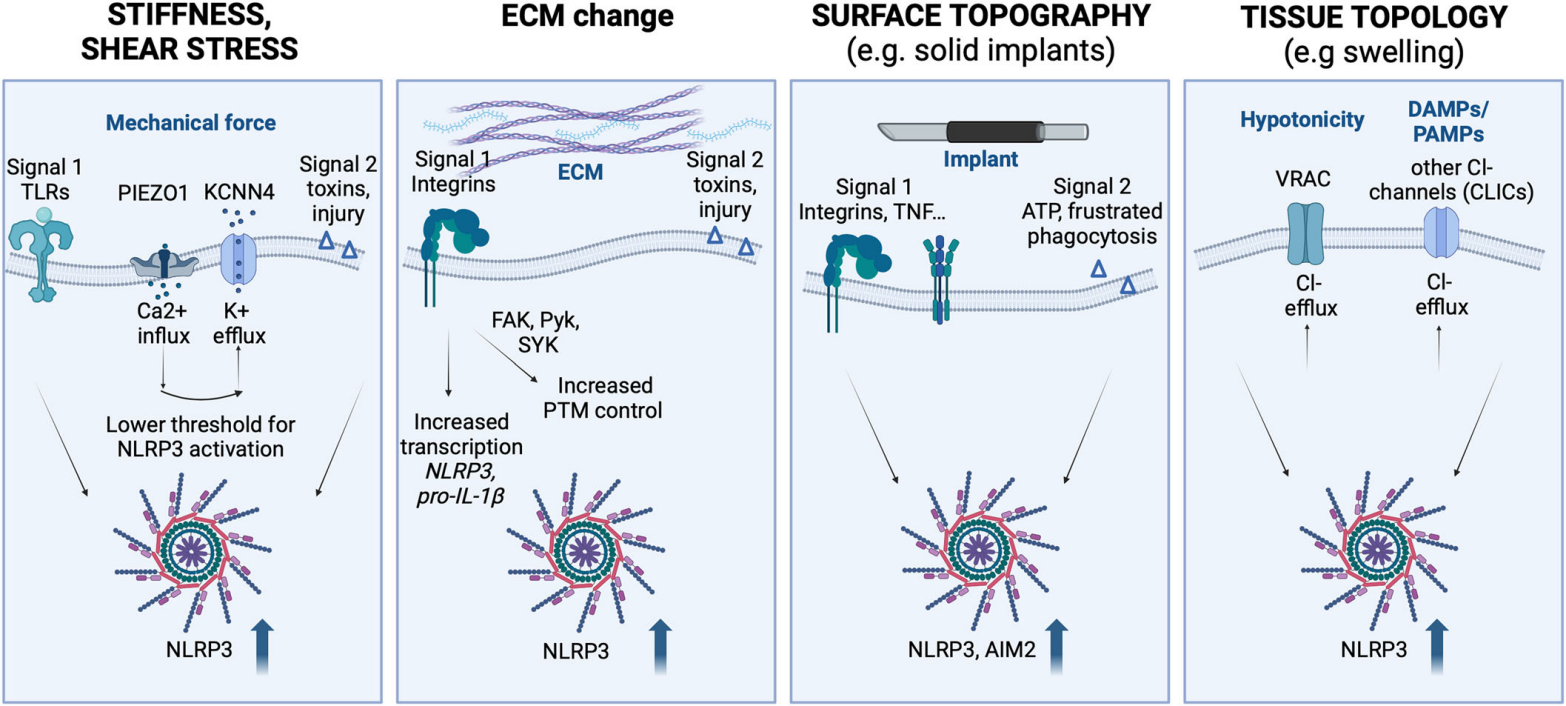
Inflammasome activity is regulated by mechano-sensing. Changes in mechanical forces exerted on cells, such as altered tissue stiffness, shear stress, the composition of extracellular matrix, the presence of foreign objects/implants or tissue/cell hypotonicity causing swelling all boost inflammasome activity. The mechanism is poorly understood but likely involves a combination of increased transcription of inflammasome genes, posttranslational control, and the control of ion (K^+^ and Cl^−^) efflux. Created with BioRender.com.

It was long appreciated that changes in intracellular mechanics affect immune receptor signalling. We and others have contributed to understanding parts of this biology (Bezbradica and Medzhitov, 2009; Fritzsche et al, 2017; Man et al, 2014). It is now clear that changes in extracellular, tissue mechanics also influence immune signalling, as these changes are sensed by immune cells which integrate mechanical signals with signals reporting the presence of infection and injury. NLRP3 inflammasome activity in macrophages is influenced by tissue stuffiness as well, and this shapes the downstream inflammatory response that macrophages elicit (Barone et al, 2022; Escolano et al, 2021; Ran et al, 2023; Vasconcelos et al, 2022). Important and emerging questions raised in this area are, how mechano-sensing of tissue mechanics alters inflammasome activity, what the upstream mechano-sensors are, which inflammasomes are affected, and if the matrix proteins upregulated in inflamed tissues contribute to inflammasome activation (Box 1). Recent efforts to answer some of these are discussed below and summarised in Fig. 1.

In need of answers

- What are the mechano-sensors that integrate changes in tissue stiffness to the activity of the inflammasome pathway, in macrophages, or the barrier cells?
- Which inflammasomes are activated by tissue mechanics and the mechanisms by which this occurs?
- How do inflammasomes induce intrinsic mechanical changes of activated macrophages (e.g. motility, shape) to allow cellular mechano-responses?
- How does activation of different inflammasomes dictate cell intrinsic mechano-adaptation, and how is it influenced by the tissue environment?
- Are tissue mechano-adaptation or mechano-inflammation affected when inflammasomes or their regulators are deleted or inhibited?

### Mechano-sensors linked with inflammasome activity

Mechano-sensors and their mechanism of action have been reviewed extensively elsewhere (Du et al, 2023; Meli et al, 2019), and are typically grouped into mechanically activated ion channels, e.g. PIEZO1 and TRPV4 (transient receptor potential V4), cell adhesion molecules e.g. integrins, cytoskeleton, and YAP/TAZ (yes-associated protein/transcriptional co-activator with PDZ-binding motif) components of the Hippo signalling pathway. Amongst these, the mechano-sensors that directly influence inflammasome activity are just beginning to emerge, and early research has focussed on ion channels, understandably, as the activity of inflammasomes such as NLRP3 is directly regulated by ion fluxes. A role of the mechanically activated ion channel PIEZO1 has been proposed, first based on the use of Yoda-1, a pharmacological PIEZO agonist (Ran et al, 2023; Syeda et al, 2015), and then confirmed by PIEZO1 deletion in the human monocyte line THP-1 cell line, or PIEZO1/2 deletion in vivo, using myeloid-specific LysMCre (Ran et al, 2023). The genetic deletion approaches are important, as the PIEZO agonist Yoda-1 can have off-target effects (Dela Paz and Frangos, 2018). A model was put forward where PIEZO1 becomes activated by the change in tissue stiffness or shear stress and triggers calcium influx into the cell. This activates a calcium-sensitive potassium channel KCNN4, to evoke potassium efflux and lower the threshold of NLRP3 inflammasome activation by nigericin, R837 or CL097, in LPS-primed THP1 cells (Ran et al, 2023). PIEZO1-mediated NLRP3 activation was particularly boosted in cells expressing CAPS-causing, NLRP3 gain-of-function mutants. Finally, PIEZO1/2 deletion in vivo reduced the pathology in NLRP3-driven disease, in a mouse model of MSU-driven gouty arthritis (Ran et al, 2023). These results generally support the link between mechano-sensing and inflammasome activation, and are in line, more broadly, with the idea that PIEZO1 activity boosts innate signalling and pro-inflammatory metabolic and transcriptional responses of macrophages, including the transcriptional upregulation of pro-IL-1β (Solis et al, 2019). TRPV4 calcium channels and integrins are also associated with the mechano-transduction in the macrophages (Lee et al, 2022; Nguyen et al, 2021), although direct signalling links between these mechano-sensors and inflammasome activity are less clear. The effector proteins of the Hippo signalling pathway, YAP and TAZ act as mechano-sensors of the cell density (Du et al, 2023). In highly dense environments, YAP/TAZ are phosphorylated and kept in the cytosol. In areas of low cell density, YAP/TAZ translocate into the nucleus and interact with the TEA domain (TEAD) family of transcription factors to promote cell growth. YAP and TAZ also move to the nucleus in areas of high extracellular matrix density and potentiate inflammatory responses in macrophages (Du et al, 2023). One study suggested that YAP promoted the inflammasome activity in mouse macrophages by promoting the stability of the NLRP3 protein, specifically by blocking the ubiquitination of NLRP3 K380 and subsequent proteasomal degradation (Wang et al, 2021). However, the exact inflammatory tissue contexts and mechanisms by which these mechano-sensors directly induce inflammasome activity in macrophages will be important to dissect in future. Other cells, particularly cells located at barrier sites, e.g. skin keratinocytes, and gut or lung epithelial cells also express a wide repertoire of inflammasome sensors and are exposed to unique mechanical stress. The mechanistic links between mechano-sensors and inflammasome activity in these cells are poorly explored.

### Tissue stiffness influences the strength of inflammasome signalling

How exactly does mechano-sensing of stiffness alter inflammasome activity, and which inflammasomes are affected? The answer to this question is likely to be complicated by variations in the experimental design used to study the mechano-regulation of innate signalling in vitro (e.g. time points, substrate properties, types of macrophages or cell lines used, readouts chosen) (Lee et al, 2022). As a consequence, some studies show a more pro-inflammatory phenotype for macrophages on stiff substrates while others show the opposite effects (Lee et al, 2022). How reflective this is of macrophages in vivo and whether these cells are similar to either monocytes or tissue-derived macrophages is unclear. Macrophages are also plastic in phenotype and are heterogeneous populations of cells in vivo, so in vitro studies need to be interpreted with caution. A reductionist analysis in vitro, however, if standardised properly in the field, will help to reveal the molecular basis and fundamental mechanisms of mechano-transduction.

To mimic tissues of different stiffness previous study cultured mouse bone marrow-derived macrophages (BMDMs) on substrates with Young’s modulus ranging from soft 0.2 kPa to stiffer 33.1 kPa. BMDMs adopted a radically different morphology, with higher circularity with more membrane ruffles on softer (compliant) substrates. BMDMs on soft substrates had enhanced NLRP3 inflammasome activity, as measured by the formation of ASC specks, and IL-1β secretion. Inhibiting actomyosin contractility diminished the differences in ASC speck formation between compliant and stiff substrates, suggesting that cytoskeletal changes affected by tissue stiffness translate directly into the signalling changes within the inflammasome complex (Burger et al, 2016; Escolano et al, 2021; Magupalli et al, 2020; Man et al, 2014).

### The extracellular matrix can prime the inflammasome activity

The extracellular matrix (ECM) is a non-cellular component of all tissues that provides a 3D structural scaffold for cells. It defines tissue stiffness; it has components that interact with cell surface receptors (e.g. integrins); and it binds to growth factors and cytokines (e.g. transforming growth factor (TGF)) that alters their bioavailability and activity (Meli et al, 2019). Matrix is made of proteins, polysaccharides, and glycoproteins, the most common matrix proteins are collagens. Tissue stiffness (defined as tissue resistance to deformation, and represented by Young’s elastic modules) is dictated by the components of the ECM, their level of expression and the type of crosslinking, the post-translational modification, and the amount of water they can bind (Griffith and Swartz, 2006). Matrix proteins are used to recreate in vitro 2D and 3D scaffolds of different stiffness. One example of how biophysical properties of the environment affect cell biology was shown by culturing stem cells in different matrix stiffness environments. These environments directed stem cell commitment towards different lineages, which were adapted to the stiffness of the tissue they populate (e.g. hard bone or soft brain) (Engler et al, 2006). In inflamed tissue, the stiffness may change because the components of the extracellular matrix change, and this alters the physical properties of tissues during disease. One example is the extracellular matrix protein Tenascin-C (TNC). TNC is expressed during embryonic development and downregulated in healthy tissues but is highly induced at sites of inflammation and trauma including for example the synovium during inflammatory arthritis (Midwood et al, 2016). Blockade of TNC reduces clinical arthritis scores and paw swelling in mouse and rat models (Midwood et al, 2009; Zuliani-Alvarez et al, 2017). As TNC is one example of an endogenous TLR activator, it is likely that upregulation of TNC, and other similar matrix proteins at peak disease, would prime the NLRP3 inflammasome expression and/or activity in macrophages. The expression atlas of matrix proteins upregulated in active disease versus resolution will bring forward matrix candidates for future studies.

Another open question is how matrix proteins regulate inflammasome activity. A few models have been proposed (Joshi and Morley, 2019). In one, activated integrins or TLRs provide signal 1 and upregulate inflammasome and pro-IL-1β expression. In the second model, matrix proteins, through integrins, directly activate kinases (e.g. FAK, SYK, Pyk (Chung et al, 2016; Gross et al, 2009; Hara et al, 2013; Lin et al, 2015)) or GTPases such as RhoA (Gao et al, 2016; Xu et al, 2014) that are involved in posttranslational control of inflammasome activation, e.g. NLRP3 and Pyrin, respectively. The role of integrins in inflammasome priming has been demonstrated in bacterial infection (Thinwa et al, 2014) and similar pathways are likely engaged by endogenous matrix proteins during tissue remodelling and inflammation.

### Change in tissue topography/adhesive geometry, such as the insertion of solid implants, triggers inflammasome activity

Extreme changes in tissue topography, such as the insertion of solid metal implants, are sensed by macrophages. The combination of local tissue damage during the implantation process and macrophage failure to phagocytose large foreign objects (frustrated phagocytosis) activates macrophages to drive acute local inflammation. The early acute inflammatory response is followed by the tissue remodelling around the implant involving fibroblast activation and fibrosis as part of the foreign body reaction (FBR), resulting in implant dysfunction (Carnicer-Lombarte et al, 2021). To understand this process, we used a nerve injury model where a microchannel device is placed into a mouse peripheral nerve injury site so that after the initial acute inflammation, axons and glia can regenerate through the channels (Barone et al, 2022). Regeneration is, however, followed by progressive fibrosis by day 28 such that the FBR forms with scar tissue extending from the channel walls, eventually compressing, and killing the axons, leading to loss of function (Barone et al, 2022). This is different from acute peripheral nerve injury (PNI) without implant placement, where transient inflammation resolves by 28 days post-surgery (Barone et al, 2022). Inflammasome pathway genes were progressively upregulated in tissues as fibrosis developed, but not in tissues recovering after injury, and this was coupled with myeloid infiltration and activation. Local inhibition of NLRP3 from the device prevented the FBR but did not interfere with tissue healing, suggesting therapeutic potential for inflammasome inhibitors to improve the performance of implantable devices (Barone et al, 2022).

Other studies also support the role of inflammasomes in the FBR using a diverse range of models. In an injection model of FBR with poly(methyl methacrylate) beads, NLRP3, ASC and caspase-1, were all required for the inflammatory response to implanted biomaterials (Malik et al, 2011). To visualise FBR, the authors used implanted 6 mm silicone discs and found that FBR capsule formation was ASC- and caspase-1 dependent but did not require NLRP3 (Malik et al, 2011). Further characterisation of FBR to poly(methyl methacrylate) bead injection revealed that both AIM2 and NLRP3 inflammasomes contributed to inflammatory and adherent properties in response to surface nano-topography and -chemistry, but that largely AIM2 was responsible for cell recruitment, collagen activity and final FBR in this model (Christo et al, 2016). These studies collectively revealed that more than one inflammasome can play a role in FBR, and that inflammasomes can be activated by a diverse range of micro- and nanoparticles during FBR.

There are two models of how inflammasomes may become activated during the FBR (Vasconcelos et al, 2022). In one model, the biomaterial causes tissue injury, this releases alarmins (e.g. ATP, DNA) which are then directly sensed by the inflammasome pathway (e.g. NLRP3 or AIM2). In the second model, inability to phagocytose larger metal implants triggers frustrated phagocytosis and inflammasome pathway activation. For either model, the size and shape of the material matter, such that larger particles and particularly those of irregular shape induce the highest levels of inflammasome activation and IL-1β release (Caicedo et al, 2013), most likely by inducing the highest level of lysosomal destabilisation during frustrated phagocytosis. Similarly, the chemical composition, and possibly the charge of the implanted material matter for inflammasome activation (reviewed in (Vasconcelos et al, 2022), however, there is no clear consensus yet on how mechanistically inflammasome pathways may be influenced by the composition of the implanted material. The success of regenerative biomedicine depends on understanding how the chemical and mechanical properties of implanted biomaterials are sensed by immune cells (Franz et al, 2011) and what immune pathways could be targeted to control different steps of FBR. Broad-spectrum immunosuppression is effective but may not be the most appropriate strategy, as acute inflammation is an essential component of the tissue repair process after implant placement. Rather targeted control of specific molecular pathways, such as inflammasomes, may become a more attractive way to control FBR.

### Changes in tissue topology, such as swelling alongside cell volume change, trigger inflammasome activity

Inflammasomes also sense tissue and cell swelling. Cell swelling through changing osmolarity leads to a volume-regulated triggering of inflammasome activity. It was reported early on, that hypotonic shock triggers IL-1β release from LPS-primed (signal 1) macrophages even in the absence of ATP (signal 2) (Perregaux et al, 1996). Later studies revealed that hypotonic cell swelling leads to the activation of Transient receptor potential (TRP) channels TRPV2 and/or TRPM7 to trigger NLRP3-dependent caspase-1 activation resulting in IL-1β processing downstream of K^+^ and Cl^−^ efflux (Boyle et al, 2013; Compan et al, 2012). Recent studies suggest that LRRC8A, a component of the volume-regulated anion channel (VRAC), plays a central role in the hypotonic induction of NLRP3 activation but is dispensable for alarmin (ATP)- or pathogen (nigericin)-induced NLRP3 inflammasome activation (Green et al, 2020). Consistent with these results, inhibitors that target chloride channels other than LRRC8A did not affect VRAC-induced inflammasome activity, but they did reduce NLRP3 activity by the classical canonical ligands, ATP or nigericin (Green et al, 2020), suggesting a general role of chloride efflux in inflammasome activation. The role of chloride efflux downstream of classical NLRP3 activators has been reported in other studies (Domingo-Fernandez et al, 2017; Green et al, 2018; Mayes-Hopfinger et al, 2021; Tang et al, 2017), and likely involves other chloride channels, such as chloride intracellular channels (CLICs). CLICs make up a family of six members (Ferofontov et al, 2020; Landry et al, 1989). They are intracellular globular proteins, that undergo extensive conformational change in response to changes in oxidative and acidic environments inside the cell. The conformational change exposes a hydrophobic patch in CLIC proteins, which allows their membrane insertion, oligomerisation, and channel formation (Al Khamici et al, 2016; Ferofontov et al, 2018; Ferofontov et al, 2020; Goodchild et al, 2011; Goodchild et al, 2009; Nesiu et al, 2019). Once in the membrane of activated macrophages CLICs amplify TLR4 signalling, inflammasome activation and IL-1β secretion (Domingo-Fernandez et al, 2017; Green et al, 2018; Tang et al, 2017). The mechanism of how exactly CLICs amplify TLR4 signalling and NLRP3 activation is not known. (Green et al, 2018). Collectively, much evidence supports the concept that changes in ion fluxes, caused by changes in tissue osmolarity, and mediated by VRAC or TRPV channels serve as important signals for inflammasome activation. Even though inflammasome activation does not require new transcription, the change in osmolarity involves complex transcriptional changes (Neuhofer, 2010), and the direct link between osmolarity and mechano-biology upstream of inflammasome activation remains to be proven.

## Inflammasome activity influences cell intrinsic mechano-responses

**Figure 2:**
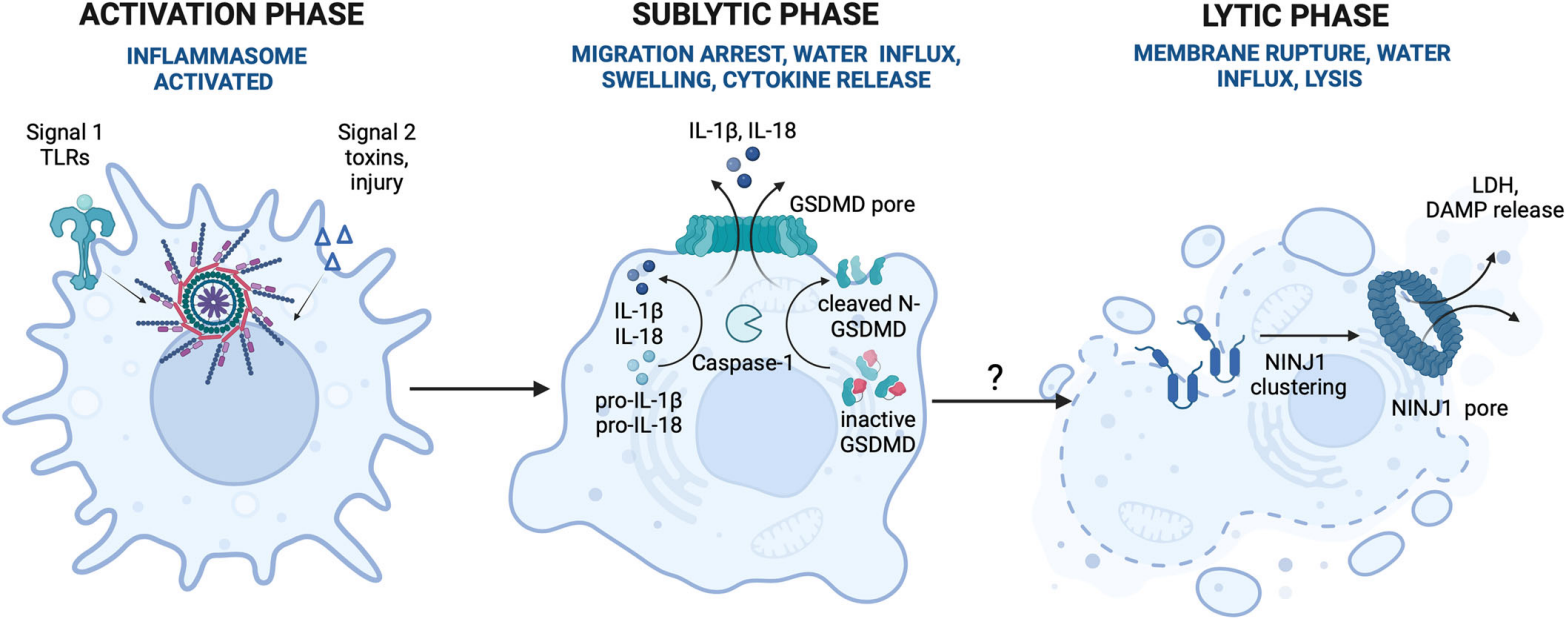
Inflammasome activity controls cell intrinsic mechano-responses. Inflammasome activity in macrophages results in the three phases of cell response. In the activation phase (left), macrophages sense the presence of pathogens and tissue injury and assemble the inflammasome complex where the effector enzyme caspase-1 is activated. In the second, sub-lytic phase (middle), active caspase-1 cleaves pro-IL-1β and pro-IL-18 into their active forms and cleaves the pore-forming protein GSDMD. Cleaved GSDMD fragment inserts into the membrane and oligomerizes to form a pore through which IL-1β and IL-18 are released. In the final, lytic phase (right), the second pore is assembled at the membrane, the NINJ1 pore, water enters the cell, and the cell undergoes lytic death and releases intracellular proteins and alarmins. Whether and how tissue mechanics affects the transition from sub-lytic, to lytic phase is not understood. Created with BioRender.com.

Once activated, inflammasomes cause a series of mechano-responses of the infected or damaged macrophages to allow their mechano-adaptation. These include changes in cellular shape, such as rounding and swelling (Chen et al, 2016), arrested motility and, usually, lytic death via the pore-forming proteins GSDMD and NINJ1 (summarised in Fig. 2). The cellular mechano-responses are important even before cell death is initiated, for example in antimicrobial defence strategies (Man et al, 2014). Our knowledge about cell-intrinsic factors downstream of ASC and caspase-1 activation that allow these mechano-responses is, however, limited. For example, what controls migration arrest, the formation, or the repair of GSDMD pores, and NINJ1 pores, how the final cell death decision is made and, importantly, how this may be influenced by the tissue mechanical environment is largely unclear (Box 1).

### Arrested macrophage motility and inflammatory death are examples of beneficial cell adaptation downstream of inflammasome activity that restricts pathogen spread during acute infections

Inflammasome activation is a dual-edged sword where excessive activity is unwanted, but it also is important in host defences. Cell death, for example, is an important defence mechanism to destroy the replicative niche for some pathogens (Doerflinger et al, 2020; Lacey and Miao, 2020; Maekawa et al, 2023). We found that even before cell death is triggered, inflammasome activation causes changes in cellular mechanics in infected macrophages to help protect the host against *Salmonella* infection. These changes included cell rounding, swelling, alteration in the actin cytoskeleton and, most importantly, arrested motility. Together with IL-1β and IL-18 secretion, this finally results in local fibroblast activation and granuloma formation with a localised fibrotic capsule that restricts bacterial spread. In inflammasome-deficient mice (NLRC4^−/−^), macrophages continued to move, granulomas failed to form, and bacteria spread through the tissue (Man et al, 2014). How inflammasome activation induces cell-intrinsic mechano-adaptation and how it regulates infection-driven fibrosis is an open question.

### Mechanism of macrophage swelling and death downstream of inflammasome activation

The inflammasome-driven activation response involves three phases (Fig. 2). The first is the activation phase, where the primed inflammasome complex is assembled, and the effector enzyme caspase-1 is recruited and activated. In the second, sub-lytic, phase active caspase-1 cleaves pro-IL-1β and pro-IL-18 into their active forms, and also cleaves GSDMD. The cleaved N-terminal of GSDMD inserts into the membrane and oligomerizes to form a pore through which IL-1β and IL-18 are released. Lytic cell death (pyroptosis) is the final phase and, along with IL-1β and IL-18 secretion, is one of the major effector functions after inflammasome activation. Importantly sometimes the inflammasome response only reaches the sublytic phase and final cell rupture does not occur (Devant and Kagan, 2023). This happens if, for example, the GSDMD pore is repaired or removed by the endosomal sorting complexes required for the transport (ESCRT) machinery (Ruhl et al, 2018). It can also occur when dendritic cells and macrophages are activated by some oxidised phospholipids released at the site of tissue damage, to trigger NLRP3-dependent IL-1β release from living cells (Evavold et al, 2018; Zanoni et al, 2016). Some myeloid cells like neutrophils can secrete IL-1β without pyroptosis, in the early hours after *Salmonella* infection (Chen et al, 2014). In most other cases pyroptosis occurs because GSDMD is processed by caspase-1 to form a pore in the cell and mitochondrial membrane (Devant and Kagan, 2023; He et al, 2015; Kayagaki et al, 2015; Shi et al, 2015; Xia et al, 2021). Elegant structural biology studies have revealed how the N-terminal of GSDMD forms a 21.5 nm pore in lipid membranes and identified that the negatively charged channel favours the transit of caspase-1 cleaved IL-1β and IL-18 (Xia et al, 2021). Recently a second pore-forming protein, NINJ1, has been identified to transduce cell lysis (Kayagaki et al, 2021). The extracellular α-helices of NINJ1 monomers when inserted in the plasma membrane polymerise NINJ1 into amphipathic filaments and/or rings with an inner hydrophobic face and an outer hydrophilic face that causes cell rupturing (David et al, 2023; Degen et al, 2023). This can be inhibited by glycine or NINJ1 selective antibodies (Borges et al, 2022; Kayagaki et al, 2023). The net result of pore formation is to drive osmotic cell rounding and swelling to occur prior to rupture. This will change the physical properties of cells including altering the membrane curvature and increasing membrane tension as well as compressing any associated structures in a tissue context. How the GSDMD pore formation transmits a signal to initiate NINJ1 filament/pore assembly remains to be identified, but a mechanism whereby changes in the cell curvature or membrane tension might contribute to this process is an attractive hypothesis.

## Inflammasome activity influences whole tissue mechano-responses

**Figure 3:**
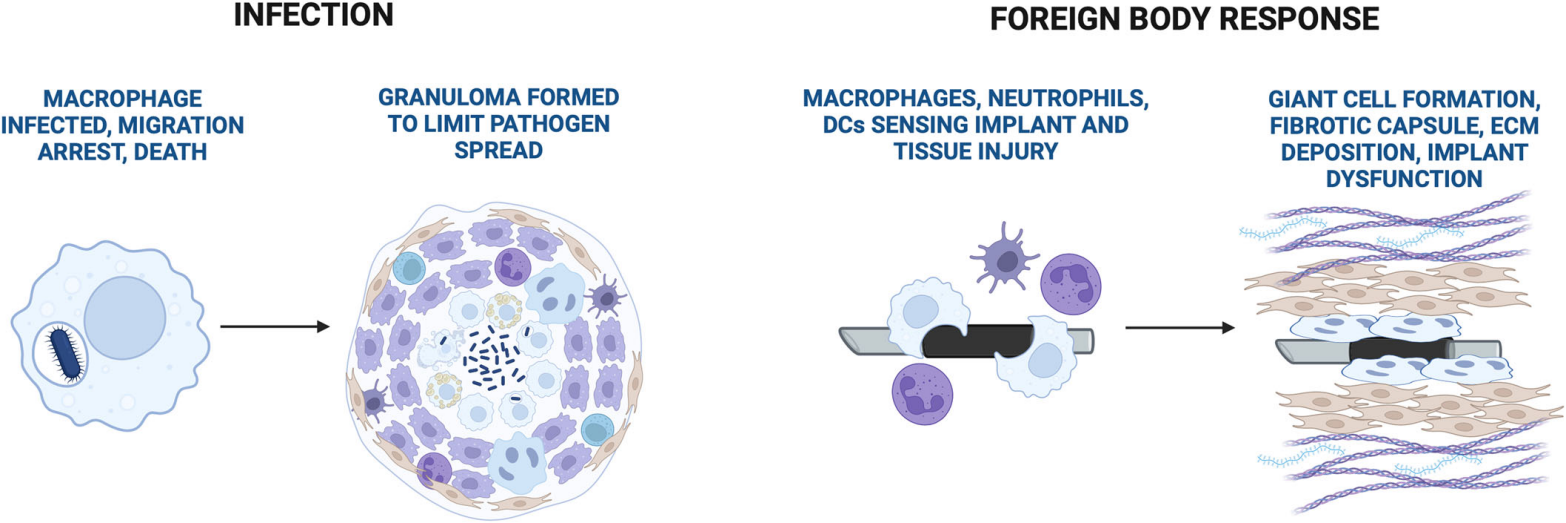
Inflammasome activity controls tissue mechano-responses. Inflammasome activity in macrophages can orchestrate the tissue mechano-responses, which can be adaptive or maladaptive. An example where inflammasomes orchestrate the tissue adaptive response is granuloma formation upon bacterial infection (left). Infected macrophages undergo motility arrest and encapsulate bacteria into the granuloma made of live and dead macrophages, neutrophils, and fibroblasts. This restricts bacterial spread. In inflammasome-deficient mice, migration arrest and granuloma formation fail and bacteria continue to spread. An example where inflammasomes orchestrate tissue maladaptive response is the foreign body reaction to solid implants (right). Macrophages, neutrophils, and dendritic cells sense the presence of a foreign implant and/or the tissue injury it caused during surgical placement. Inflammasomes are activated in response to tissue injury, by ‘frustrated phagocytosis’ (inability to phagocytose the large implant) and/or the physical properties of the implant. To encapsulate the implant, macrophages form multinuclear giant cells, neutrophils get recruited and fibroblasts are activated. Fibroblasts deposit an extracellular matrix around the implant and form the fibrotic capsules. As a result, the implant is disabled and dysfunctional. In mice where the device locally releases an NLRP3 antagonist, the foreign body reaction is reduced, and the implant remains functional. Created with BioRender.com.

Recent work described that the inflammasome pathway within macrophages orchestrates fibrotic capsule formation as a whole-tissue response to some infections and solid implants (Barone et al, 2022; Christo et al, 2016; Malik et al, 2011; Vasconcelos et al, 2022) and we summarised emerging models in Fig. 3. The mechanistic basis for inflammasome-driven tissue mechano-adaptation in vivo is not well understood. Some of the questions and examples are discussed below: how tissue mechano-adaptation, mechano-induced inflammation and fibrosis are affected in vivo when inflammasome genes are genetically deleted or pharmacologically inhibited?

### Fibrosis is tissue maladaptation that occurs downstream of sustained inflammasome activation

Formation of a fibrotic capsule around a solid implant occurs as the FBR develops which can be visualised by smooth muscle actin (aSMA) staining (Barone et al, 2022). This is prevented by coating implants with the inflammasome inhibitor MCC950, or the broad-spectrum immunosuppressant dexamethasone (Dex) prior to surgery. Importantly, only MCC950, but not Dex, still allowed axon regeneration through the implant, suggesting that local inhibition of inflammasome activity, but not generalised immunosuppression prevents the FBR, whilst retaining tissue (in this case axon) repair. We confirmed that inflammasome inhibition prevents fibrosis using an independent model of FBR to subcutaneous implants (Barone et al, 2022) suggesting a more general role of inflammasome activity in the development of fibrosis. Fibrosis is a major unsolved problem and we provided evidence that persistent inflammasome activation contributes to tissue maladaptation and fibrotic capsule formation (Barone et al, 2022). These results are in agreement with earlier studies where the FBR was induced after injection of large sterile microspheres into the peritoneal cavity of wild-type mice, which triggered similar myeloid infiltration, activation of NLRP3 and AIM2 inflammasomes and fibrotic capsule formation (Christo et al, 2016; Malik et al, 2011; Vasconcelos et al, 2022).

In a broader sense, inflammasome contribution to tissue fibrosis is a problem that goes beyond FBR and is particularly well described in the context of liver fibrosis upon viral infection, non-alcoholic steatohepatitis (NASH), or alcoholic liver disease (ALD) (Alegre et al, 2017), and in lungs upon mechanical ventilator-induced lung inflammation and subsequent fibrosis (Kuipers et al, 2012; Lv et al, 2018). In all these cases NLRP3 deletion or inhibition in mice reduces fibrosis and tissue pathology. How inflammasomes contribute to fibrosis in any of those conditions is unclear, but one may envision that the process involves at least two models. In one model, there is direct activation of fibroblasts (or other tissue-resident stromal cells) by IL-1β or alarmins released by activated macrophages. In another model, inflammasome-dependent IL-1β and alarmins induce chemokines that recruit neutrophils into the tissue, to drive acute inflammation. This is typically followed by neutrophil apoptosis, removal of apoptotic cells by macrophages, TGFβ secretion and TGFβ-mediated fibroblast activation and induction of fibrotic programmes. Which of these models drive FBR remains to be tested.

### Recruited inflammatory myeloid cells are the main source of inflammasome activity and IL-1β production with fibroblasts being one of the main responders to IL-1β in inflamed tissues

NLRP3 and many other inflammasome proteins are expressed most highly in myeloid cells (Guarda et al, 2011), which we also saw in the FBR (Barone et al, 2022), and in many available scRNA-Seq data. For example, scRNA-Seq analysis of the human (Zhang et al, 2019) and mouse arthritic synovium showed low inflammasome and IL-1β expression in tissue-resident macrophage subsets (MERTK+, FOLR+, TREM2+, TIMD+ subsets and TREM2+, STMN1+, RETNLA+, LYVE1+, ACP+ subsets). The levels are high, however, in recruited pro-inflammatory macrophages (CSFR2, CCR2+ subsets), particularly as the disease develops by day 5 in a mouse model of serum transfer-induced arthritis. Other groups have identified recruited neutrophils as the main source of IL-1β at peak inflammation in vivo (Chen et al, 2014; Friedrich et al, 2021; Friedrich et al, 2019; Khoyratty et al, 2021). Interestingly, fibroblasts are the cells with the highest IL-1R expression in many inflamed tissues (Friedrich et al, 2021; Friedrich et al, 2019), and human fibroblasts respond to IL-1β stimulation to induce cytokine secretion (Demarco et al, 2022). Furthermore, recent work suggested that within the tissue (e.g. inflamed synovium) there are unique micro-environments where IL-1β-driven pathological macrophage–fibroblast crosstalk may happen (Smith et al, 2023). We thus hypothesise that chronic inflammasome activation in myeloid cells, as seen in FBR, arthritis or colitis, produces local IL-1β, which, together with other local tissue micro-environment signals, contributes to pathological fibroblast activation, tissue dysfunction and, in some cases, fibrosis.

## Conclusions

The mechanistic basis for mechano-sensing and mechano-responses in immunology is not well understood (see also Box 1). To determine the importance of inflammasomes in mechano-inflammation, how mechano-sensing triggers inflammasome activity in response to changes in tissue/substrate stiffness, transient extrinsic mechanical load, or a combination of mechano-stimuli needs to be determined. The availability of inflammasome reporter lines/mice (Tzeng et al, 2016), of inflammasome-sufficient or -deficient cells/mice (Kovarova et al, 2012; Kuida et al, 1995; Mariathasan et al, 2004; Rauch et al, 2017; Tenthorey et al, 2020; Wang et al, 1998; Wang et al, 2017), and live imaging and single-cell approaches will make the answers to these questions technically possible.
